# Randomized Feasibility Trial of Teleyoga versus In-Person Yoga for Treating Chronic Musculoskeletal Pain in Veterans

**DOI:** 10.21203/rs.3.rs-6265068/v1

**Published:** 2025-06-23

**Authors:** Louise Mahoney, Shweta Pahade, Heidi Mahoney, Korrine Fitz, Audrey Keaney, Kelly Parker-Bridges, Jasmin M. Francisco, Annie Lau, Rita Hitching, Beth Walker, Arushi Gupta, Booil Jo, Jessica A. Lohnberg, J. David Clark, Peter J. Bayley

**Affiliations:** War Related Illness and Injury Study Center, VA Palo Alto Healthcare System; War Related Illness and Injury Study Center, VA Palo Alto Healthcare System; War Related Illness and Injury Study Center, VA Palo Alto Healthcare System; War Related Illness and Injury Study Center, VA Palo Alto Healthcare System; War Related Illness and Injury Study Center, VA Palo Alto Healthcare System; War Related Illness and Injury Study Center, VA Palo Alto Healthcare System; War Related Illness and Injury Study Center, VA Palo Alto Healthcare System; War Related Illness and Injury Study Center, VA Palo Alto Healthcare System; War Related Illness and Injury Study Center, VA Palo Alto Healthcare System; VA Palo Alto Health Care System; War Related Illness and Injury Study Center, VA Palo Alto Healthcare System; Stanford University; VA Palo Alto Health Care System; VA Palo Alto Health Care System; War Related Illness and Injury Study Center, VA Palo Alto Healthcare System

**Keywords:** yoga, teleyoga, chronic pain, musculoskeletal pain, veteran, feasibility

## Abstract

**Background:**

Chronic pain impacts approximately 20% of the United States adult population and 50–75% of Veterans. It is treatment-resistant, and medications include the risk of addiction or overdose. The VA is promoting complementary and integrative health (CIH) for use along with existing treatments. Yoga can provide effective treatment for many health problems, including pain. Some of the challenges of providing yoga in healthcare include barriers such as space, time, and transportation. We studied the feasibility of conducting a randomized controlled trial (RCT) treating Veterans with chronic musculoskeletal pain with yoga delivered online versus in-person.

**Methods:**

We developed a yoga protocol for treating chronic musculoskeletal pain online using online cohorts (n = 9, 15) with chronic musculoskeletal pain. Optimal treatment parameters were established. The resulting yoga protocol consisted of 12 weekly 75-minute classes with home-practice on 5 non-class days/week. The second phase of the study established the feasibility of conducting an RCT comparing in-person and online yoga. Thirty-four participants (30 male) were randomized to in-person (n = 16) or online yoga (n = 18). Measures were collected at baseline and end-of-treatment.

**Results:**

We successfully met our Veterans participant recruitment goals for the study. Furthermore, the retention rates were 83% for teleyoga and 68% for in-person yoga, which exceeded our a priori feasibility target of 65%. Protocol adherence was 8.62 classes (71.8%, range = 2–12) in the teleyoga group and 9.25 classes (77.1%, range = 4–12) for in-person yoga, again exceeding our feasibility rate of 65%. Treatment satisfaction was measured using a 33-item questionnaire where each item was rated on a 0–4-point scale. Average treatment satisfaction was 3.2 in the teleyoga group and 3.6 in the in-person group, exceeding the feasibility target of ≥ 2. There were no serious adverse events. Yoga fidelity was assessed by scoring 20% of the classes and was 91% overall. Analysis of secondary outcome measures showed that only the teleyoga group experienced a statistically significant reduction in pain.

**Conclusion:**

It is feasible to conduct an RCT comparing teleyoga to in-person yoga to treat chronic musculoskeletal pain in Veterans. Treatment may provide a reduction in pain severity and pain interference.

**Trial registration:**

clinicaltrials.gov NCT04074109, August 29, 2019

## Background

Chronic pain is a debilitating condition with high societal and economic costs ([1], [2]). Pharmacological pain management which includes opioids and other analgesic medications, can be dangerous and/or ineffective after long-term use ([3]). Consequently, increased efforts are being made to find nonpharmacological treatment options for the management of chronic pain. Indeed, pain is the condition for which adults in the United States most often use complementary and integrative health (NHSR#98–2016). For these reasons, rigorous research on complementary and integrative health (CIH) for pain is a public health priority.

The VHA National Pain Management Strategy, initiated in 1998, issued directive 2009–053 which included standards of pain management and endorsed implementation of “Pain as the 5th Vital Sign” in all inpatient and outpatient clinical settings”. The use of yoga to treat chronic pain has been endorsed by many organizations including the National Institutes of Health (NIH), the American College of Physicians and the VHA (Veterans Health Administration) ([4], [5]). The VHA has been a leader in offering CIH services at medical facilities. A 2017–2018 survey of CIH approaches ([6]) used in 196 Veterans Administration (VA) medical centers and community-based outpatient clinics (CBOC) revealed that CIH services were widespread in VA with yoga being provided at 131 of those VA facilities. Indeed, the provision of VA care via telehealth to increase access to those living far from VA medical facilities is a VA priority.

Unfortunately, not everyone has access to therapeutic yoga classes. This is particularly true for those living in rural areas or without resources to pay for yoga classes. There can be other barriers to practicing yoga, including travel costs and time, health conditions, bad weather, lack of transportation, and family responsibilities. One solution to overcome these barriers is to provide yoga at home using internet-based technology (“teleyoga”). Teleyoga became increasingly popular during the recent pandemic as yoga studios quickly ramped up offerings online with some yoga studios switching to an online only model. Teleyoga is an innovative, potentially effective approach for treating chronic pain in Veterans ([7]). However, research evaluating the acceptability or efficacy of teleyoga is very limited. Our earlier pilot study indicated that teleyoga was safe and had similar efficacy to in-person yoga for treating several common conditions in Veterans, including chronic musculoskeletal pain ([8]).

Despite the widespread use of teleyoga, there is a lack of fully powered randomized controlled trials (RCT) demonstrating its efficacy to treat chronic pain. Conducting an RCT in patients to treat chronic pain could be challenging for several reasons. For example, for reasons of safety and communication, questions need to be addressed about maximum class size, type of yoga, and methods of communication (e.g., verbal instruction vs. demonstration). In addition, participants randomized to either group might have a preference of one group to another and drop out if they are not randomized to their preferred group. Additionally, in-person participants may receive benefit from practicing yoga along with other Veterans in the same room but might find it difficult to travel to the study site and may drop out more frequently than their online peers.

The primary aim of this study was to demonstrate the feasibility of conducting a randomized controlled trial comparing at-home teleyoga to in-person yoga for the treatment of chronic musculoskeletal pain. This aim was addressed in two phases. In the first phase we developed a yoga intervention suitable for treating chronic musculoskeletal pain online. Particular attention was given to technical and communication challenges of providing yoga via video conferencing software and optimizing visibility and audibility. In the second phase we conducted a pilot RCT using the methods and techniques developed in Phase 1. The primary feasibility outcomes included rates of recruitment, retention, protocol adherence, fidelity of treatment delivery, participant satisfaction and adverse events. Feasibility was determined by comparing these outcomes to benchmarks from similar studies ([9], [10]) and setting a priori benchmarks based on our previous yoga study ([11]).

### Phase 1

The study was funded by the National Center for Complementary and Integrative Health (NCCIH), the study protocol was approved by the Institutional Review Board (IRB) of Stanford University and the study adheres to CONSORT guidelines.

The goal of Phase 1 was to develop a teleyoga protocol suitable for treating Veterans with chronic musculoskeletal pain. Multiple aspects of protocol design were considered including 1) Equipment needs of participants and teachers; 2) Choice of videoconferencing software; 3) Determination of a maximum class size for safety and efficacy purposes; and 4) Establishing recruitment methods, 5) Participant satisfaction feedback (scoring an average of ≥2 (range of 0–4) on the Satisfaction Questionnaire).

#### Methods – Phase 1

##### Inclusion Criteria:

1) Being a Veteran; 2) Medical clearance for participation by VA primary care provider; 3) Experiencing musculoskeletal pain with a severity between ≥4 to <9 on a 0–10 scale (the Defense and Veterans Pain Rating Scale v2.1 (DVPRS) [12]) for ≥6 months; 4) Ability to travel to the study site at VA Palo Alto; 5) English literacy; 6) wireless internet access (WiFi) and a space for practicing yoga at home.

##### Exclusion Criteria:

1)Current participation in another clinical trial; 2) Back surgery within the last 12 months; 3)Back pain related to a specific underlying cause, disease, or condition; 4)Baseline pain <4 or >9 on the DVPRS; 5) Unstable, serious coexisting medical illness; 6)Unstable, serious coexisting mental illness; 7) Attended or practiced yoga ≥1 time in the past 12 months; 8) Active current suicidal plan or intent.

##### Recruitment Methods:

We posted flyers throughout the VA Palo Alto Healthcare System (VAPAHCS), set up recruitment tables at the VAPAHCS weekly farmers market, and contacted Veterans who had previously indicated they wished to be contacted for future research projects. We recruited a total of 25 participants with chronic musculoskeletal pain for Phase 1 of the study (see Table 1 for detailed list of recruitment sources).

##### Videoconferencing Equipment and Software:

We tested several different video conferencing software packages during Phase 1 (VA Video Connect, Webex, and Zoom) and found a HIPAA compliant version of Zoom provided the most flexible viewing options and remained stable in low bandwidth conditions. We chose an omni-directional desktop microphone system for the teachers (eMeet Luna wireless speakerphone chosen for price and performance). To increase the teachers’ view of the class we tested a large (31 inch) external computer monitor and several computer projection systems and settled on a short throw, high lumen projector (ViewSonic Inc., DLP Projector, PS600X). The teachers were also provided with a webcam with remote controlled zoom and positioning features (Logitech BCC950 ConferenceCam).

##### Participant Equipment:

Prior to treatment, each participant received an iPad, a desktop iPad holder, a yoga mat, a yoga strap, 2 yoga blocks, 2 yoga blankets and the modified printed homework packet. The packet included step-by-step instructions with photos indicating options for pose adaptation.

#### Measures - Phase 1

The primary outcome for Phase 1 was to see if we could recruit Veterans to participate in yoga online and to see if they would practice yoga online with at least moderate satisfaction. Phase 1 was also used to determine optimal class size, as a test run for the online homework log created for the study and as an opportunity to adapt a 10-week in-person yoga protocol to a 12-week online and in-person program.

##### Treatment Satisfaction:

Satisfaction of treatment was measured using customized version of the Multi-Dimensional Treatment Satisfaction Measure (MDTSM) ([13]). The questionnaire contained 33 questions scored on a on a 5-point Likert scale [from 0 (very unsatisfied) to 4 (very satisfied)]. Treatment satisfaction assessed participant experience with the yoga classes, instructors, printed material, yoga’s contribution to improvement in function and pain relief. We added three free text questions to allow participants to provide information on what they found most bothersome, suggested improvements, and general comments, bringing the total count to 36 questions. For each participant the mean score from all 33 Likert ratings was calculated and for the assessment of feasibility, we set the group mean target score on treatment satisfaction to ≥2, representing neutral or positive satisfaction.

##### Homework Log:

Homework practice was collected weekly using an online log that collected binary data (home practice yes/no?) and optional reporting on length of practice and what yoga elements they used (freeform text). Participants were encouraged to enter zero minutes of practice in the log rather than not complete it at all.

##### Yoga Protocol:

We adapted the yoga protocol developed for a clinical trial to treat chronic musculoskeletal pain in Gulf War Veterans ([11]). The protocol and homework packet were expanded from 10 weeks to 12 weeks and modified to maximize clarity and safety for online delivery and remove the need for in-person assistance. Since the original protocol had been designed as an in-person protocol, modifications focused on simplifying the instructions and enhancing our skills at providing clear and concise verbal instruction. The original teleyoga protocol was specifically designed to treat a broad range of musculoskeletal pain types including bromyalgia-like pain, back pain, and joint pain. It was developed from a Hatha yoga/Krishnamacharya tradition but included adaptations for the individual using yoga props and household items such as chairs and walls. The protocol included yoga tools such as controlled breathing techniques (pranayama), yoga postures (asana), hand gestures (mudra), vocalization, and meditation (dhyana) (See Supplemental Table 9, Additional File 1 for an outline of the yoga protocol). All yoga elements were offered in a manner suitable for participants without prior yoga experience. Each element was selected for its potential to enhance awareness of habits that might lead to increased pain such as improper ergonomics in daily activities and habits that might improve pain such as improvements in posture, muscle strength and flexibility, as well as autonomic nervous system function. Some elements targeted pain by focusing on the musculoskeletal system (postures/asana) and others targeted pain using an eastern medicine lens which has at its core a concept life force (prana) and the balance and communication between systems using a network of pathways called “nadis”. The yoga elements were chosen to affect movement of prana by opening and balancing the energy centers and pathways without referring to prana or nadis. The intervention and homework were fully manualized, including demonstration of the use of props such as blocks, straps, chairs, walls, and blankets. Participants were asked to practice yoga for 15–20 minutes on 5 non-class days/week.

During Phase 1 we hired four additional yoga teachers with a minimum of 200-hour yoga teacher training and current registration with Yoga Alliance (to ensure continuing education requirements are up to date). Yoga staff met weekly during the study over Zoom to unify teaching methods and re ne the yoga protocol. Details on the hiring and training process are provided in Additional File 2.

#### Procedure

Participants completed baseline assessments in person (cohort 1) or online (cohort 2) during the week prior to starting the yoga sessions. The group classes met online once per week for 75 minutes and were conducted in 2 cohorts composed of 9 and 16 individuals. One participant was lost to follow-up after baseline assessment. Participants were asked to record whether they completed their homework practice (yes/no) and provide optional information on what they did for practice. Classes were taught by one yoga instructor.

#### Results - Phase 1

The primary aim of Phase 1 was to establish the parameters and materials for a teleyoga treatment protocol suitable for treating Veterans with chronic musculoskeletal pain. The resulting yoga protocol consisted of 12 weekly 75-minute classes with home-practice on 5 non-class days/week, with a maximum class size of 5. This protocol was used in Phase 2.

The yoga class experience was moderately well received as indicated by the Satisfaction Questionnaires (mean =3.25, SD= .67, range=1.48–3.94). Optimal class size was determined to be 5 based on ability of the instructor to view participants sufficiently well to ensure safety, comfort, and stability in the postures, the hallmark of a yoga practice.

### Phase 2

#### Methods – Phase 2

##### Inclusion/Exclusion Criteria:

We utilized the same inclusion and exclusion criteria same as Phase 1.

##### Recruitment Methods:

Due to the pandemic, Phase 102 recruitment shifted to contact by mail. We conducted a search of electronic health records of patients at the Palo Alto VA Hospital to identify Veterans likely to have chronic musculoskeletal pain using a list of 61 ICD 10 and 12 ICD 9 codes which identified over 9000 Veterans. We sent recruitment letters with interest response cards to 5089 Veterans who met criteria of living within a 20-mile range of the main hospital facility. Potential participants either phoned the study coordinator or return a completed interest card.

**Figure 1:**
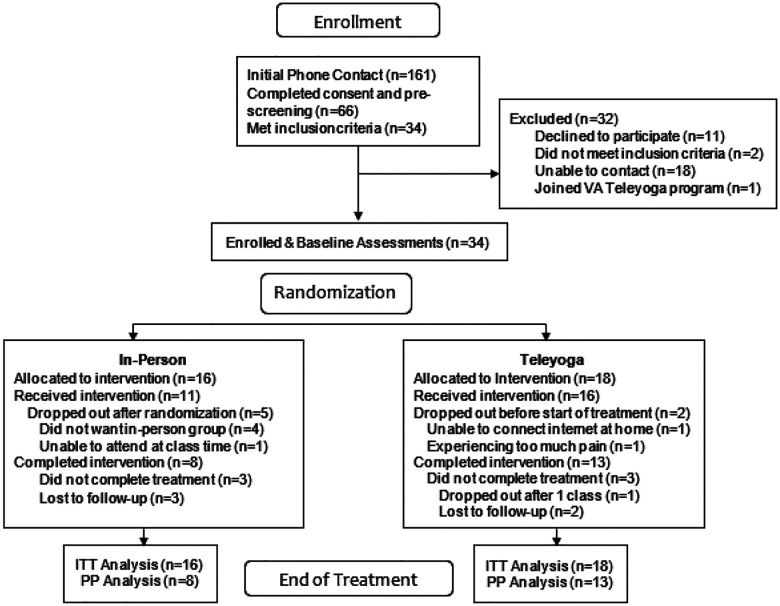
Phase 2 Consolidated Standards of Reporting Trials (CONSORT) Flow Chart

Veterans who were interested in the study were consented and screened by phone. Pain levels were assessed using DVPRS. Eligible participants were enrolled in three study cohorts of up to 10 participants each. Participants completed baseline assessments online up to 1 week before starting treatment after which they were randomized in equal numbers into in-person and teleyoga groups using random number tables provided by the study statistician and implemented by the study coordinator. EOT assessments were completed online within one week of ending treatment. We recruited 34 participants (Table 1). A total of 34 Veterans with chronic musculoskeletal pain were recruited and randomized to a 12-week program of yoga offered in-person (n=16) or via telehealth (n=18) (See Fig. 1).

Recruitment details can be found in Table 1.

##### Videoconferencing Equipment and Software:

We utilized an HIPPA compliant version of Zoom as determined from Phase 1. Each Veteran received an iPad and tripod iPad holder as well as a Bluetooth speaker when needed. The tripod iPad holder provided the ability to adjust angle of viewing for seated, standing, and supine postures.

#### Primary (Feasibility) Measures

##### Treatment Protocol Adherence.

We operationalized treatment adherence as the percentage of randomized participants attending of ≥65% of the 12-week intervention (i.e., attending at least 8 of the 12 sessions) as assessed by weekly attendance logs, as minimum rate for feasibility ([10], [9] [14, 15]).

##### Recruitment Rate.

We set the recruitment rate as the ability to complete recruitment within the project timeline of one year.

##### Attrition Rate.

Attrition rate was set as the number of randomized participants who dropped out of treatment during the study. The benchmark attrition rate of <35% was chosen for adequate feasibility based on larger studies involving yoga treatment for Veterans with chronic musculoskeletal pain that have typically reported attrition rates of approximately 36%−40% by the end of treatment ([16, 17]).

##### Treatment Satisfaction.

Satisfaction of treatment was measured using the MDTSM ([13]) as described above. For the assessment of feasibility, we set the group mean target score of ≥2, representing neutral or positive satisfaction.

##### Treatment Fidelity.

Video recordings of the teachers during the yoga session were made for all sessions. A qualified yoga instructor scored 20% randomly selected sessions from each cohort using a fidelity checklist created from the yoga manual. Yoga instructors did not score their own sessions. Scored elements included beginning of class centering, breath practices (pranayama), yoga postures, and meditation ([18] [19]). We set an a priori benchmark for minimum competency of ≥95% of treatment components based on a previous RCT using a similar in-person yoga protocol ([11]).

##### Missing Data Rate.

We calculated the aggregated missing data rate at both timepoints for all participants. We used a benchmark missing data rate of ≤15% for adequate feasibility based on a reasonable estimate of data collection.

##### Adverse Events.

A Data Safety Monitoring Committee was convened for this project, who reviewed safety concerns every 6 calendar months.

#### Secondary Outcome Measures

##### Pain, Enjoyment and General Activity Scale (PEG):

Pain was measured using the PEG 3-item self-report measure [20] that assesses pain severity and pain interference. It includes one pain severity question (average pain on a 0–10-point scale with 0 being no pain and 10 the worse pain) and two pain interference questions (enjoyment of life, and activity (also 0–10-point scales) and a total score which is the average of the 3 scores.

##### Brief Pain Inventory-Short Form (BPI-SF):

Participants completed this 17-question instrument on pain severity and pain interference over the past 24 hours ([21]).

##### PROMIS Pain Interference - Short Form 6b V1.0:

Participants completed a 6-question instrument that asked how much pain had interfered with activities and enjoyment of life in the past 7 days [22].

##### BDI-II:

Depression was measured using the Beck Depression Inventory, a 21-question instrument for measuring symptoms of depression [23].

#### Exploratory Outcome Measures

##### Medication Use:

A self-report custom questionnaire requesting use, dose, frequency and start date of prescription medications and supplements was given at baseline and end of treatment. Reporting was freeform and reliant on participant recall.

##### Other Therapy:

A self-report custom questionnaire requesting use, dose, frequency of other therapies utilized for pain was given at baseline and end of treatment.

##### Homework Log:

As described above, the online log collected yes/no information whether a daily practice was done and optional description of the practice. Based on feedback from Phase 1, we created online videos to guide home practice in addition to the printed homework packet. The videos were published unlisted on Youtube.com.

#### Yoga protocol – Phase 2

The yoga intervention developed in Phase 1 (see above and Supplemental Table 9, Additional File 1) was used in Phase 2 and was administered either in-person or as a live class over Zoom. Each class was taught by one instructor with an assistant instructor in the room or online to provide support as needed (see Figure 1, Additional File 1). Instructors alternated roles as teacher or assistant during the 12-week session. For those who had difficulty hearing, a Bluetooth speaker was an adequate solution provided for enhanced hearing ability at home.

Due to COVID-19 restrictions, both the instructors and students in the in-person group were asked to wear masks and remain 6 feet apart during class. The teleyoga classes were conducted without masks. All classes were recorded for fidelity assessment.

### Statistical Analysis

We employed descriptive statistics to calculate the primary outcome feasibility metrics using means and standard deviations for the continuous, and frequencies and percentages for categorical variables.

For the intention to treat (ITT) analysis of the secondary outcome measures of pain and depression, we employed standard linear mixed effects modeling (e.g., [24]; [25]) to estimate outcome changes from the baseline to post intervention assessment within each group and their differences between the groups. We used maximum likelihood estimation with robust standard errors implemented in Mplus version 8.6 (Muthén & Muthén, 1998–2017). Data points that were missing due to subject attrition were handled assuming that data were missing at random ([26]) conditional on observed information. In our mixed effects analyses, the change (slope) in the outcome was modeled as the key dependent variable predicted by the treatment assignment status. In all analyses, we used the nominal significance level (alpha=.05, two-tailed). Given the preliminary nature of the study we did not adjust the significance level for multiple testing. The results based on longitudinal mixed effects modeling are presented in Table 4. For within group changes, effect sizes are calculated by dividing the estimated slope by the baseline sample standard deviation. For group differences (efficacy) in changes, effect sizes are calculated by dividing the estimated slope difference by the baseline sample standard deviation pooled across the two groups.

## Results

The two treatment groups did not differ significantly in baseline characteristics except that there were no female participants in the teleyoga group (see Table 2: Demographics, and Supplemental Table 1, Additional File 1 for baseline measure characteristics).

### Recruitment

We met the recruitment criteria of being able to recruit a total of 50 participants (20 in Phase 1, 30 in Phase 2) within the time frame of the study.

### Attrition Rates

Attrition from the in-person group was 8 (50%), and 3 (17%) from the teleyoga group. Reasons for attrition are shown in Fig. 1 and additional attrition data is shown in Supplemental Table 2, Additional File 1.

### Protocol Adherence

**Figure 2:**
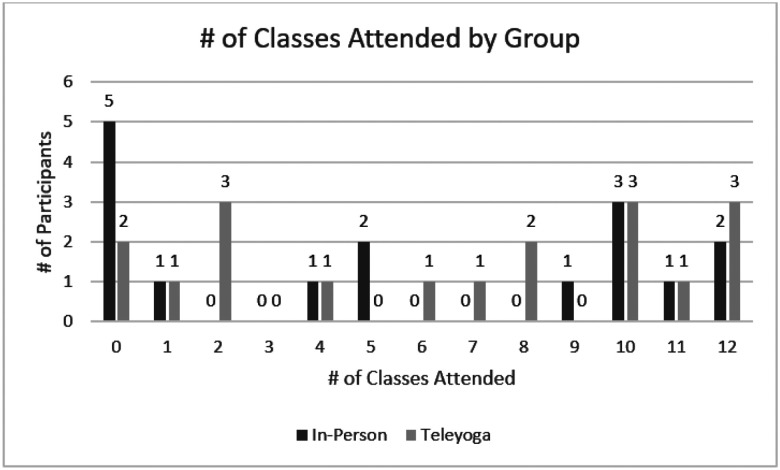
Total Attendance by Group

Protocol adherence was defined as the percentage of randomized participants who attended ≥ 65% of the treatment classes and was 60% and 75% in the in-person and teleyoga classes respectively. Figure 2 provides a graphical representation of class attendance.

Distribution of total number of classes attended in each treatment group.

### Participant Satisfaction

Satisfaction in both groups was generally in the higher range –between 3 and 4 on the 0–4 scale – except on the question of discomfort from yoga which scored more towards the mid-range indicating a mild amount of discomfort for some participants (Table 3). In general, the participants were satisfied with the yoga research program and found it helpful for relief of pain. When asked about functional improvement and pain relief because of yoga, the in-person group gave more attribution to yoga for their improvement than the teleyoga group despite the larger improvement in symptoms in the teleyoga group.

Three of the questions asked for freeform written feedback about what aspects of the program were most bothersome, suggestions for improvement, and general comments. Here are some of the comments we received:

### General feedback

“Loved it and appreciated the pain relief. Thank you”.

I have been taking a maximum dose of Gabapentin for neuropathy for years. The calming breathing exercises has reduced the twitching and jerking so much so that I have reduced my Gabapentin intake gradually to about 75%.

The thing I enjoyed was going into yoga sessions in person and going home to practice what learned in class, then returning to following week learn something new and discussing what problems I encountered from my homework plus get questions answered. As I mentioned before having a current videos online would be great. The instructors were all GREAT. I know that my pain will be with me, these yoga exercises have made the pain management easier for me, not to mention that have been able to more sleep and relaxing. I have set 3–4 days a week for my yoga and during my daily activities I try incorporating breathing exercises and some poses when possible. Now that I know that I do my yoga standing or sitting is great. The only negative thing was that in person is over.

Instructors were very good and enjoyable to work with

### Suggestions for improvement

The Apple iPad were nice but the screen as too small to see the instructors’ demonstrations. I frequently had to get off the chair or mat and get up close to the screen.

### Bothersome

difficulty hearing instructions

“Too far to travel. We could use this in the Monterey clinic”.

### Fidelity of Yoga Protocol Delivery

Protocol fidelity for was 91% for the in-person and 90% for the teleyoga classes as shown in Supplemental Table 3, Additional le 1. Overall fidelity was 91%.

### Missing Data

The overall missing data rate was 8.25%. In the teleyoga group, missing data ranged from 4.2% at baseline to 4.9% at EOT. In the In-person group, missing data ranged from 8% at baseline to 15.9% at EOT. Missing data involved the Promis-6b scale only. See Supplemental Table 4, Additional File 1 for details.

### Secondary Outcome Measures - Symptom Improvement

Table 4 shows the estimated within- and between-group changes in secondary outcome measures based on longitudinal mixed effects modeling. In the teleyoga group, most outcomes except depression (BDI-II) showed clinically meaningful improvements with medium to large effect sizes (d = 0.42 to 0.84). Among those, the improvements in pain (PEG, BPI severity, and BPI interference) were statistically significant. In the in-person group, most outcomes showed less improvements than in the teleyoga group except in depression. The largest improvements were shown in pain (PEG average, d = 0.59) and depression (BDI-II, d = 0.35). None of the outcomes showed statistically significant improvements in the in-person group. The largest differences between the two groups were shown in pain severity (BPI severity, d = 0.45) and pain interference (PROMIS6b, d = 0.46), although they were not statistically significant due to our limited sample size. Overall, teleyoga showed promising results in most key outcomes. In the teleyoga condition, pain severity and interference (PEG, BPI, PROMIS6b) showed considerable improvement that were even greater than in the in-person condition.

We also looked at symptom improvement in the PP group (those who received the intervention and completed EOT assessments) and found the trend toward greater improvement in pain severity and interference in the teleyoga group (n = 13) and greater improvement in depression in the in-person group (n = 8) (see Supplemental Table 5, Additional File 1).

### Exploratory Measure Results

#### Homework and Homework Logs

Two participants in teleyoga and one in in-person yoga completed all 12 weeks of the homework log. As for the homework practice itself, 9 (69%) participants in the teleyoga group did at least one practice session during the 12-week study (M = 5.2, SD = 4.4) and 5 (63%) participants in the in-person group reported at least one practice session during the study (M = 4.8, SD = 4.3). Supplemental Table 6, Additional File 1 provides a summary of the homework practice metrics.

#### Medication Usage

To analyze, medications were grouped together by their medical classification (analgesic, opioid, statins, hormones etc.) then these classifications were grouped by their purpose (pain relief, diabetes treatment etc.). For each medical classification, we reported the number of individuals taking one or more medications of that type at baseline. The most common medications were anti-hypertensive medications (29%), over the counter pain medications (26%) and statins (24%). The teleyoga group reported higher usage of medications at baseline (Supplemental Table 7, Additional File 1).

#### Non-pharmacological Therapies

Thirty-eight percent of participants did not list any non-yoga therapies at baseline and the next highest reported therapies were Exercise (18%), Physical Therapy (12%), Chiropractic (12%), and Acupuncture (12%). The baseline data provided insight into the general level of activity of participants prior to the yoga treatment protocol (Supplemental Table 8, Additional File 1).

## Discussion

Overall findings indicated that it is feasible to conduct an RCT on yoga vs. teleyoga in Veterans with chronic musculoskeletal pain. We met the benchmark rates of recruitment (ability to recruit 50 participants within the timeline of the study), attrition (≤ 35%), protocol adherence (≥ 65% in both groups), and treatment satisfaction (≥ 2 on a 0–4 scale). Although total attrition met the feasibility benchmark (≤ 35%), the In-Person group attrition was high (50%). We will need to over recruit in a large clinical trial in order to meet the benchmark of ≤ 35%. We had predicted that teacher protocol fidelity would be at least 95% based on results from a previous study; however, that study relied on self-report of fidelity rather than an independent review. In this feasibility trial, we relied on others to rate the fidelity from class recordings. Based on this experience we feel it would be reasonable to set fidelity targets of 80–85% for a successful trial of a yoga protocol and that our overall fidelity rate of 91% is above that target benchmark. Overall, we met the feasibility benchmark on missing data (≤ 15%) and had zero adverse events.

We assessed medication usage and therapies to determine how they changed across treatment however the assessment instrument utilized was awed as it relied on self-report before and after the trial based on memory. When a medication was missing in the post-treatment assessment, we could not accurately determine if it was omitted by mistake or if the participant had stopped taking the medication. In a larger RCT we will utilize a more systematic approach to reliably assess medication usage before and after the intervention. We think such information is useful and worth some effort to collect in a future RCT – particularly as it relates to analgesic medications including opiates.

Both in-person and teleyoga groups reported above average satisfaction with the yoga classes. However, it is interesting to note that the perception of yoga’s contribution to pain relief was lower in the teleyoga group despite the indication that they received greater improvement. This may be due to the small sample size, but one possible explanation could be that the in-person group developed a closer bond with the instructors and therefore “felt” that yoga was more responsible for symptom improvement. In both groups there was always one instructor leading the class and one instructor offering suggestions for modifications and use of yoga props; however, in the in-person group, the assisting instructor could help to place the prop rather than offer a verbal suggestion. In the teleyoga group there were four rotating instructors whereas there were only two instructors for the in-person group. Perhaps there was more opportunity for a therapeutic bond to form in the in-person class with the same two instructors for the entire 12 weeks. A larger study might address that potential treatment difference by using the same two instructors (one leading and one assisting) for both telehealth and in-person classes for each cohort.

Several factors may have contributed to the differences in pain and depression outcomes between the two groups. The in-person group was attending class during the time when there was a masking mandate at the VA – for both the teachers and the participants. The telehealth participants did not need to wear masks. Since a significant portion of the yoga protocol involved controlled use of breath – or yoga breathing practices –the effects may have been altered for those using masks.

The difference in outcome in the depressions scores might be explained by the greater opportunity for there to have been a group cohesion/social interaction effect in the in-person yoga group than in the teleyoga group. The in-person group might have also received a type of behavioral activation by coming to an in-person class that was missing in the teleyoga group. Behavioral activation is a known intervention for depression. A Larger trial can help determine if meeting in-person contributes to improvement in symptoms of depression. Incorporating time for social interaction into both groups might serve to improve group cohesion in both groups.

Additional lessons learned regarded hearing loss which is one of the more prevalent service-connected disabilities in Veterans [27]. During Phase 1 of the study, we tested numerous microphones and found that both a wireless over the ear mic and an omnidirectional mic worked well for most participants. We also discovered that providing Veterans with an inexpensive Bluetooth speaker greatly enhanced the ability to hear and follow the instructor.

We asked participants to practice for at least 15 minutes on 5 non-class days and complete an online homework log to record their weekly practice. It was optional to report what they did for practice. Only a few participants reported that they utilized the homework videos in the logs, but many Veterans reported to the instructors that they used the videos and found them useful. Supplemental Fig. 2, Additional File 1 provides a glimpse of video usage during the time frame of the study and illustrates that the videos were used much more than reported in the online logs. Future studies could develop this technology to gather individual data on video usage and homework participation. As mentioned above, each Veteran also received a printed guide for home practice. Many participants reported using the guide and finding it useful. We suggest future studies explore methods for improving home practice adherence and monitoring.

## Conclusion

Overall, we were able to develop a protocol suitable for online delivery of yoga and test the feasibility of conducting an RCT to compare online vs. in-person yoga. Each of the primary feasibility targets were met (recruitment, attrition, adherence, satisfaction, missing data, treatment fidelity, and adverse events). Secondary outcome measures indicated that yoga provided significant improvements in symptoms of depression in both in-person and online treatment groups. Additionally, the teleyoga group showed statistically significant improvements in pain severity and interference. With the rapid expansion of online yoga, it is more important than ever to provide scientifically sound evidence for its benefits for treatment of chronic musculoskeletal pain, and these results provide the foundation to successfully conduct a RCT for this purpose.

## Supplementary Material

Supplementary Files

This is a list of supplementary files associated with this preprint. Click to download.

- Additionalfile3.docx
- Additionalfile2.docx
- additionalfile3.docx

## Figures and Tables

**Table 1:** Recruitment Methods and Response

Recruitment Type	Phase 1 Enrolled only	Phase 2	Eligible	Enrolled
**Direct mail from medical record data search** 5089 letters sent. Response cards/phone calls received	0	No Response=4581 (90%) Returned mail=372 (7%) Card: Not Interested=124 (2%) Card: Interested=43 (<1%) Calls: Interested=44 (<1%) Calls: Not Interested=0	43	31
**Flyers** (VA Palo Alto bulletin boards)	5	5	4	2
**Clinician referral**	2	4	3	1
**Fellow Veteran referral**	1			
**Announcement in phone-based meditation**	5			
**Internet** “Volunteer for Research” section	0	2	0	0
**WRIISC newsletter**	0	1	1	0
**Research contact list**	10	1	0	0
**Unknown**	2	36	1	0
**Total**	25	5089	52	34

**Table 2:** Demographics

Demographics					
	Phase 1 (n = 24)	Phase 2 (n = 34)	Total	Phase 2	
	N (%)	N (%)	%	In-Person	Teleyoga
**Gender**					
Male	11 (46)	30 (88)	71%	12	18
**Race**					
Asian	5 (21)	5 (15)	15%	1	4
White	12 (50)	23 (68)	68%	11	12
Black or African American	4 (17)	2 (6)	6%	2	1
Unknown		1 (3)	3%		1
Other		4 (12)	12%	3	1
Indian or Alaskan Native	3 (12)				
Multi-race		2 (6)	6%		
**Ethnicity**					
Hispanic or Latino	5 (21)	5 (15)	15%	3	2
**Age**					
**Mean Age (STD)**	58 (9.33) *(range = 32–73)*	61.06 (17.21) *(range = 32–89)*		62 (15.09)	61 (18.90)
**Marital Status**					
Married	6 (25)	22 (65)	65%	10	12
**Employment Status**					
Employed Full Time	3 (12.5)	9 (26)	26%	4	5

**Table 3:** Participant Satisfaction*

Question Categories (question #s)	In-Person (n = 8) Mean (SD)	Teleyoga (n = 13) Mean (SD)
Accessibility and Utility of Yoga (1–7)	3.50 (0.78)	3.04 (0.98)
Overall Yoga Experience (8)	3.88 (0.33)	3.62 (0.62)
Attitude Toward Yoga (9–10)	3.88 (0.33)	3.31 (1.10)
Rating of Yoga Instructor(s) (11–16)	3.75 (0.43)	3.74 (0.52)
Rating of Printed Information (17–20)	3.59 (0.55)	3.21 (0.79)
Rating of Yoga Classes (21–25)	3.78 (0.52)	3.22 (0.18)
Functional Improvement Attributed to Yoga (26–29)	3.38 (0.70)	2.69 (0.77)
Discomfort from Yoga (30)	2.75 (1.09)	2.50 (0.96)
Pain Relief Attributed to Yoga (31–33)	3.21 (0.82)	2.64 (0.82)
Combined Average (STD)	3.57 (0.31)	3.17 (0.49)

* Results from a 36 item Multi-Dimensional Treatment Satisfaction Measure.

**Table 4:** Estimated within-group and between-group changes based on longitudinal mixed effects modeling (ITT)

		Teleyoga (n=18)	In-person (n=16)	Group Difference
*PEG average*	Change	−1.446	−1.146	−0.300
	p-value (2-tailed)	0.003	0.126	0.737
	Effect size*	−**0.82**	−**0.59**	−0.16
*BPI severity*	Change	−1.311	−0.515	−0.796
	p-value (2-tailed)	<0.001	0.369	0.239
	Effect size*	−**0.84**	−0.26	−**0.45**
*BPI interference*	Change	−0.988	−0.563	−0.426
	p-value (2-tailed)	0.020	0.457	0.624
	Effect size*	−**0.46**	−0.24	−0.19
*PROMIS6b total*	Change	−2.639	0.354	−2.993
	p-value (2-tailed)	0.099	0.867	0.260
	Effect size*	−**0.42**	005	−**0.46**
*BDI2 total*	Change	−0.333	−2.286	1.952
	p-value (2-tailed)	0.754	0.116	0.278
	Effect size*	−0.06	−0.35	0.33

* Effect sizes are in Cohen’s d. Effect sizes that are 0.4 or greater are bolded.

## Data Availability

The datasets used and/or analyzed during the current study are available from the corresponding author on reasonable request.

## Abbreviations

PEG: Pain, Enjoyment of Life and General Activity
BPI-SF: Brief Pain Inventory-Short Form
Promis6b: Patient-Reported Outcomes Measurement Information System 6 question form on pain interference
CIH: Complementary and Integrative Health
VHA: Veterans Health Administration
NIH: National Institutes of Health
VA: Veterans Administration
CBOC: Community-based Outpatient Clinics
RCT: Randomized Controlled Trials
SQ: Satisfaction Questionnaire
IRB: Institutional Review Board
HIPAA: Health Insurance Portability and Accountability Act
MDTSM: Multi-Dimensional Treatment Satisfaction Measure
ICD: International Statistical Classification of Diseases
COVID-19: Coronavirus Disease 2019
VAPAHCS: VA Palo Alto Health Care System
DVPRS: Defense and Veterans Pain Rating Scale
Wi-Fi: family of wireless network protocols based on the IEEE 802.11 family of standards
TY: Teleyoga
IP: In-Person
EOT: End of Treatment
BDI-II: Beck Depression Inventory
ITT: Intention to Treat
WRIISC: War Related Illness and Injury Study Center
PP: Per Protocol
PI: Principal Investigator
